# Intravenous administration of puppy deciduous teeth stem cells in degenerative valve disease

**DOI:** 10.14202/vetworld.2016.1429-1434

**Published:** 2016-12-16

**Authors:** Soontaree Petchdee, Sarunya Sompeewong

**Affiliations:** 1 Department of Large Animal and Wildlife Clinical Science, Faculty of Veterinary Medicine, Kasetsart University, Kamphaengsaen, Nakhorn Pathom 73140, Thailand; 2 Kasetsart University, Veterinary Teaching Animal Hospital, Kamphaeng Saen, Thailand

**Keywords:** dental pulp stem cells, dogs, heart failure

## Abstract

**Aim:**

The objective of this study is to investigate the improvement of heart function in dogs with chronic valvular heart disease after puppy deciduous teeth stem cells (pDSCs) administration.

**Materials and Methods:**

20 client-owned dogs with degenerative valvular heart disease underwent multiple intravenous injections of allogeneic pDSCs. Dogs were randomly assigned to two groups: (i) Control group (n=10) with standard treatment for heart failure and (ii) group with standard treatment and multiple administrations of pDSCs (n=10). Electrocardiography, complete transthoracic echocardiography, thoracic radiography, and blood pressure were recorded before and after pDSCs injections for 15, 30 and 60 days.

**Results:**

Post pDSCs injection showed measurable improvement in left ventricular ejection fraction, American College of Veterinary Internal Medicine (ACVIM) functional class significantly improved and improved quality of life scores were observed. In the control group, there were no significant enhancements in heart function or ACVIM class.

**Conclusions:**

This finding suggests that pDSCs could be a supplement for valvular heart disease treatment.

## Introduction

Degenerative valve disease (DVD) is the most common of cardiac diseases in small breed dogs; valve degeneration is the loss of structural integrity of the heart valve leaflets caused by infirmity of collagen and elastic tissue of the valves. Cardiac remodeling secondary to heart valve degeneration may also contribute to damage the heart muscle and the progression of the disease appears to be unpredictably [1]. Ventricular dilation and dysfunction is a common complication of DVD. Clinical appearances of DVD include exercise intolerance, coughing, difficulty breathing, and weakness. Diagnosing can be done through several different procedures such as auscultation of the chest, radiographic imaging (X-rays), electrocardiography, echocardiography (ECHO), biomarker testing such as N-terminal proatrial natriuretic peptide and platelet function testing [2,3]. Although ECHO is a common noninvasive technique for diagnosing valve degeneration in dogs, ECHO established fully characterize the structure and function of the heart, new imaging method such as cardiac imaging resonance (CMR) is expected to provide more certainly measurement of heart function than ECHO [4]. Therapeutic considerations of DVD were determined according to the American College of Veterinary Internal Medicine (ACVIM) guidelines. Drugs such as diuretics, angiotensin-converting-enzyme (ACE-I), and positive inotropes provide the basis of pharmacological treatment of heart failure. However, treatment of the valve degeneration is only alleviating its progression. Further study is still required for this disease intervention.

Stem cell transplantation becomes widely studied for therapeutic approaches in the field of regenerative medicine [5-7]. Mesenchymal stem cells (MSCs) can be isolated from a variety of organs and tissues such as bone marrow, brain, skin, hair follicle, skeletal muscle, and dental pulp [8]. Recently, stem cells therapy has shown therapeutic efficacy for regenerating damaged myocardium [9-11]. However, the clinical use of several stem cells has been controversial and limited due to the ethical concerns [12,13]. Dental pulp stem cells are not only derived from a source which is noninvasive, avoided the ethical issues and also able to supply enough cells for clinical application. These dental pulp stem cells have MSC qualities, including the capacity for self-renewal and multilineage differentiation potential [14-17]. Dental pulp stem cells might provide a potential role of the treatment for heart failure resulting from mitral degeneration.

The objective of this study was to determine whether puppy deciduous teeth stem cells (pDSCs) could be used as a new therapeutic approach for chronic heart valve disease in veterinary patients.

## Materials and Methods

### Ethical approval

This study was approved by the Ethical Comm- ittee for Animal Experiments, Kasetsart University, Thailand.

### Cells preparation

Cells were harvested and collected from the puppy teeth and then cultured in Dulbecco’s modified Eagle’s medium (Sigma-Aldrich, St. Louis, MO, USA) supplemented with 10% fetal bovine serum (Invitrogen, Gaithersburg, MD, USA) and 1% penicillin/streptomycin at 37°C, 5% CO_2_. Cells at 80% confluency were harvested via trypsin-ethylene diamine tetra-acetic acid treatment. Cells (pDSCs) at passages 1, 2 and 3 were characterized by intracellular flow cytometry (Santa Cruze Biotechnology, CA, USA). Cells were freshly prepared in phosphate buffered saline (PBS) and suspended in 2 ml PBS. The MSCs adhered to plastic culture dishes and formed fibroblast-like colonies and cells surface marker of Stro1 were analyzed as previously described [18,19].

### Enrollment criteria

Dogs with heart murmur of moderate and high intensity and had atrioventricular valve regurgitation with the stage of heart failure in C according to ACVIM classification system were recruited to this study [20]. Dog’s owners provided consent form and questionnaires with quality of life questions prior, 30 and 60 days after the study was begun. Randomized double-blind was used in this study, dogs were randomly divided into two experimental groups, consist of Group 1 (control): Dogs given standard treatment for heart failure including diuretics, ACE-inhibitor and PBS administration (n=10); Group 2: Dogs given standard treatment and pDSCs (1 × 10^6^) injected intravenously at day 0 (start the treatment) and then at days 14 after pDSCs therapy initiation (n=10).

### Clinical evaluation

In a blind clinical study, all dogs were subjected to clinical evaluation consisted of physical examination, routine hematology, blood biochemistry, thoracic radiography, and blood pressure measurement. After the clinical evaluations, the variables from questionnaires were scored according to the parameter as shown in Table-1. The modified ACVIM functional class was used to grade the severity of heart failure.

**Table-1:** Scoring protocol for quality of life evaluations.

Variables	Score	Clinical sign
Demeanor	1	Alert, responsive
	2	Mildly depressed
	3	Moderately depressed
	4	Minimally responsive
	5	Unresponsive
Exercise intolerance	1	Inactive, only get up to eat and drink or urinate
	2	Less active than normal, moved around a few times per day, avoided long walk
	3	Able to move around but not able to fully exercise, the ability to run was reduced
	4	Able to move around and fully exercise
Coughing	1	None
	2	A few times a week
	3	A few time a day
	4	Frequently during the day
Appetite	1	Increased
	2	Normal
	3	Decreased
	4	Markedly decreased

### Thoracic radiography

The 2 orthogonal views of chest radiography were performed. Vertebral heart score (VHS) was measured in the left lateral view. Lung pattern was recorded and evaluated on all dogs at day 0 and day 30 and day 60 after the initiation of treatment.

### ECHO examination

All dogs underwent a transthoracic ECHO with continuous electrocardiogram monitoring (General Electric Medical System, vivid5s, Germany). The measurement was performed in parasternal long, short axis views and apical four-chamber view when dog was on the right and left lateral recumbency with no sedation. ECHO was evaluated before and after pDSCs administration by one skillful sonographer. ECHO images were stored for offline analysis. Mitral regurgitation jet area was determined using continuous-wave color flow Doppler ECHO using the left apical 4-chamber view.

### Electrocardiography (ECG) examination

The 3-min surface ECG recording was performed with all dogs in right lateral recumbency. ECGs was recorded and analyzed on all dogs at day 0 and day 30 and day 60 after the initiation of treatment.

### Treatment

Cells (pDSCs) were suspended in 2 ml PBS and administered into cephalic vein in dogs in the pDSCs-treated groups. All dogs in the treatment group were scheduled to receive two intravenous administrations of 1 × 10^6^ cells of pDSC per kg of body weight at day 0 and day 14 after the first pDSCs administration. A similar administration of 2 ml of PBS was given to dogs in control group.

### Statistical analysis

Data are expressed as mean±standard deviation. Statistical analysis between control and treatment group were assessed using paired t-test (Graphpad prism version 5). A p=0.05 or less was determined for statistically significant.

## Results

### General characteristics

Clinical characteristic was shown in Table-2. Dogs in control and pDSCs-treated group had an average age of 11.13±0.58 and 11.20±0.93 (range: 8-15 years old) and average weight was 6.24±0.93 and 6.62±0.57, respectively. The dogs were 7 poodles, 5 mixed breeds, 4 shih-tzus, 2 miniatures, 1 yorkshire terrier, and 1 pomeranian. All dogs were in heart failure Stage C. Heart rate and arterial blood pressure in all dogs remained stable throughout the study. In pDSCs-treated group (n=10), the cardiac rhythm of dogs in ECG examination include sinus tachycardia in 6 dogs (60%), respiratory sinus arrhythmias in 2 dogs (20%), and premature supraventricular beats in 2 dogs (20%). In control group (n=10), 7 dogs (70%) presented with sinus tachycardia and 3 dogs had premature supraventricular beats (30%).

**Table-2:** Baseline and clinical characteristics.

Variables (mean±SEM)	Control	pDSCs-treated	p
Mean age (years)	11.3±0.58	11.2±0.93	0.36
Mean weight (kg)	6.24±0.56	6.62±0.57	0.36
VHS	13.42±0.38	13.57±0.54	0.33
Sex (M/F)	7/3	5/5	

VHS=Vertebral heart score, M=Male, F=Female, SEM=Standard error of mean

### Radiographic findings

The average VHSs from radiograph of dogs were not statistically different after pDSC administration. VHS in control and pDSCs-treated group were 13.42±0.38 and 13.57±0.54, respectively. Two dogs had pulmonary edema, 9 dogs had interstitial lung pattern, and 9 dogs had mixed lung pattern. The VHSs were not different after pDSCs administration.

### ECHO findings

**Figure-1:**
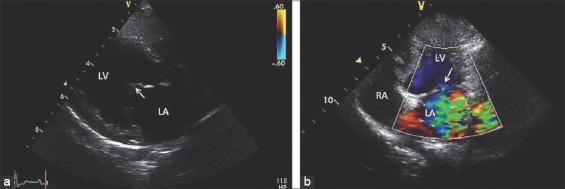
Echocardiographic images from right parasternal long axis view of the mitral valve degeneration of the dog. The arrow represented the thickening of mitral valve leaflet (a). In (b) showed left ventricular inflow tract, arrow indicated the point of mitral regurgitation jet, LA: Left atrium, LV: Left ventricle and RA: Right atrium.

**Figure-2:**
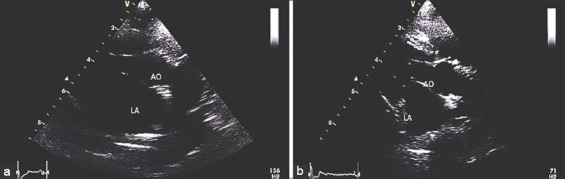
Parasternal short axis and M-mode plane of the left ventricular of the dog before (a) and after (b) administration of puppy deciduous teeth stem cells. LA:Ao; diameter of left atrium and aortic root ratio.

**Figure-3:**
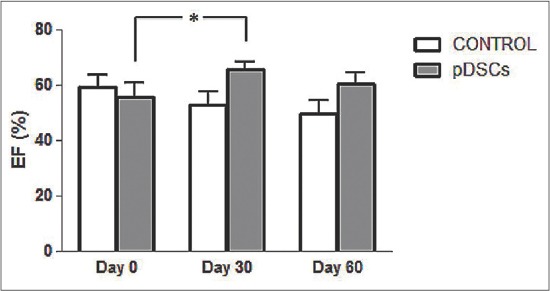
Ejection fraction in dogs with valve degeneration before (day 0), 30 and 60 days after puppy deciduous teeth stem cells administration (day 30 and day 60) compared with the control group, *p<0.05.

**Figure-4:**
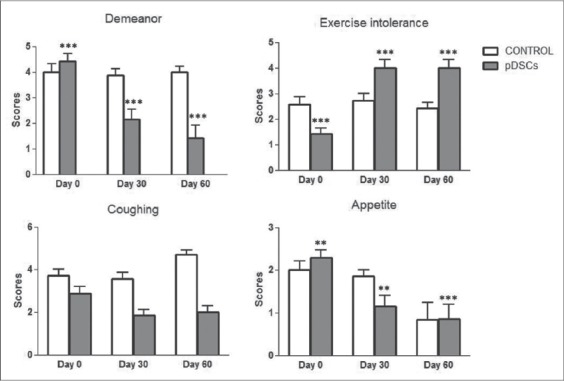
Life quality in dogs with valve degeneration before (day 0) and after 30 days and 60 days of puppy deciduous teeth stem cells administration (day 30 and day 60), ***p<0.0001 and **p<0.001.

Results were shown in Table-3. The example of ECHO features of mitral valve degeneration was shown in Figure-1. A decreases of diameter of left atrium and aortic root ratio were observed in pDSCs-treated group as shown in Figure-2. The interventricular septal end diastole, interventricular septal end-systole, left ventricular posterior wall end systole, and left ventricular posterior wall end diastole were not significantly different after pDSCs administration. However, measurable improvement in left ventricular ejection fraction (Figure-3), ACVIM functional class significantly improved and improved quality of life scores were observed until 60 days after pDSCs administration (Figure-4). Ventricular diameter (LVIDd and LVIDs) in pDSCs injected group was also improved but did not change significantly.

**Table-3:** Percentage changes of echocardiographic parameters (mean±SEM) 60 days after pDSCs administration compared with the control group.

Percentage change (%)	Control	pDSCs-treated
LVIDd	−2.63±6.23	−9.74±3.14
LVIDs	−1.35±9.24	−10.70±8.54
EF	−5.67±5.79	8.57±6.18
FS	−6.28±7.84	11.15±7.59
LA/Ao	−4.21±2.89	−5.11±3.2

LVIDd=Left ventricular end diastolic diameter, LVIDs=Left ventricular end systolic diameter, EF=Ejection fraction, FA=Fractional shortening, LA: Ao=Diameter of left atrium and aortic root ratio, SEM=Standard error of mean

## Discussion

This is the first study to show the application of pDSCs to treat chronic valvular heart disease in dogs. We provided evidence that multiple pDSCs administered through an intravenous route exert effective therapeutic without complications. Many investigations have been directed at how to optimize the therapeutic effectiveness of stem cells. Most of cell transplant studies suggested that stem cells therapy required a large quantity of cell (more than 100 million cells) to show an improvement of heart function. However, many investigators showed that less amount of MSCs can exert therapeutic effects and be independent from the number of cells homing to the targeted organ [21,22]. In this study, we are beginning to investigate whether intravenous administrations of 1 × 10^6^ of pDSCs per kilogram body weight can exert beneficial therapeutic effects in dogs with chronic valvular disease. It is well known that MSCs therapy can be attributed mainly to the release of paracrine and cytokine factors [23]. The mobilization of stem cells by paracrine and cytokines requires about 1 week to attain a peak response [24]. In addition, transplanted cells will be in the circulation for about 48 h to release the chemotactic agents and nine out of ten cells will eventually die by 2 weeks after transplantation [25]. Thus, in this study, repeated injection was performed after 2 weeks of the first injection. However, the exact dosage of stem cells transplantation is not clearly understood. The basic issue such as the optimal dosage and time course of stem cells therapy will require the formulation of a new standard for therapeutic use of stem cells in cardiovascular diseases. Many unresolved questions about the dosage and number of injections of stem cells for clinical application are still open for the future research.

Results from this study showed that administration of pDSCs significantly improves heart function, quality of life, decreases LA dimensions as shown in Figure-2. Transthoracic ECHO is the diagnostic test in our present study. Echo is a noninvasive imaging technique to investigate the cardiac function and morphology. However, several factors would tend to result in underestimations of heart volumes using ECHO such as errors from the heart motion or arrhythmias during measurements. CMR is increasingly applied for the heart function measurement. CMR might provide the images of the heart that more precise than ECHO [26]. ECHO versus CMR comparative study should be performed to confirm the effects on the heart function after pDSCs administration. Other limitations of this study, we did not examine the histological of the valve leaflets and the serum level of inflammatory markers after the treatment. However, mitral regurgitation jet was improved in pDSCs treated group. It might indirectly suggest that pDSCs-treated group might provide the improvement of the valve lesion. It is still unclear whether therapeutic effects are the result of differentiation of stem cells into specialized cell types or preserving their self-renewal function. In this study, we also did not perform the study to track stem cell movement. However, the previous study has shown that dental stem cells can migrate to the damaged tissue, growth factors and paracrine factors such as SDF-1, HGF and VGEF from dental pulp stem cells are the keys that involved as chemotactic and homing of stem cells to the damaged tissue/cells [27,28]. Paracrine effects are a possible mechanism that enhances neovascularization, reduces inflammation and is involved in the heart remodeling. Many studies have identified the paracrine and growth factors that may help to repair the heart valve tissue such as tumor necrosis factor α, vascular endothelial growth factor, fibroblast growth factor, insulin-like growth factor, and interleukin-6. These growth factors would induce the angiogenesis, anti-apoptotic, anti-fibrotic and correlate with the left ventricular geometric change during the remodeling processes [29,30].

However, understanding the paracrine mechanism of pDSCs for regenerative therapy requires further studies to support our finding. Furthermore, a long-term following up is needed to study the mechanism of cells therapeutic actions [31,32]. As can be observed in our study, improvement in left ventricular systolic function in dogs treated with pDSCs might be due to the paracrine effects. Although immunohistochemistry examination to quantitative scoring of paracrines type to indicate the amount of paracrine content has not been investigated in this study, indirect results showed left ventricular dimension decreasing in ECHO imaging. In this study, the quality of life scores were used to determine the therapeutic effects of pDSC and it is essential to have scoring methods that allow for reduced variability between various dogs and owners. Questionnaire of life quality provides a way for the owner who is most familiar with the dog to provide insight into the dog’s abilities in the home environment. In this study, the quality of life scores at day zero, the score appeared difference between control group and pDSC-treated group. However, the quality of life scores could answer by the owners blinded to the treatment, we cannot exclude some degree of subjectivity in the evaluation from questionnaire, and this is a limitation of this study. Our findings seem to support that pDSCs administration might be used to decelerate the progression and alleviate the symptoms of heart failure resulted from heart valve degeneration. Although a further study is required for ascertaining of pDSCs treatment in heart diseases, this finding opens an alternative approach for the heart valve degeneration in veterinary animals.

## Conclusions

This finding suggests that pDSCs could be used as an alternative treatment for valvular heart disease in dogs.

## Authors’ Contributions

Both authors contributed the relevant work, SP: Principle investigator, drafted and revised the manuscript. SS: A research coordinator. Both authors read and approved the final manuscript.
